# The Norwich Collection of Calculi

**Published:** 1888-02-04

**Authors:** 


*(To be continued.)*

### THE NORWICH COLLECTION OF CALCULI.

NEXT to that of the Royal College of Surgeons in London, the most complete collection in the world of stones removed from the human subject is that which is preserved in the museum of the Norfolk and Norwich Hospital. There are three reasons for this—the first being that in this county of Norfolk cases of renal and vesical calculus are more numerous than in any other part of the country; secondly, that the hospital, during the hundred odd years of its existence, has been singularly fortunate in having secured the services of a long line of skilful surgeons, who have made a special study of the disease, and each of whom has vied with his predecessor in his desire to increase the medical fame of the city and the hospital; and thirdly, that the hospital—in the words of Mr. Cadge, its senior surgeon—"presents, in the accuracy with which its books have been kept, a model for all hospitals." Certainly, the museum in its present hands will suffer from no want of thoughtful and even affectionate care and attention. Its curator, Mr. T. W. Crosse—himself a stone-surgeon of repute, and the son of a father no less distinguished in the treatment of the same disease—would not be the man to allow the fame of the museum to decay; and he is assisted by Mr. W. G. Crook, whose heart, it is easy to see, is in his work. Every stone which has been removed from patients in the hospital, either during life or after death, becomes the property of the hospital. Here accordingly are nearly one thousand two hundred specimens, with the name and age of every patient, of the surgeon by whom the operation was performed, and what was the result of it. Every circumstance which can attend the operations of lithotomy or lithotrity—the former consisting in the removal of the entire stone, and the latter in its being crushed into small pieces before it is taken away—is in this way made accessible to the student, who is thus put into the possession of the united experience of some half-dozen men whose names have become familiar as household words in the treatment of this disease.

The first of the long line was Benjamin Gooch, whose work on surgery is a standard book to this day. He was a lithotomist of distinction, and one of the founders of the hospital, to which he was consulting surgeon, but without active duties. His name, therefore, does not often appear as an operator on the cards in the cases, but his collection of amputating instruments is preserved. A medical student of to-day might scoff at them, no doubt; but they did good work in their time, and it is fitting that the hospital which Gooch helped to establish should piously preserve these memorials of him.

On the centre table is a remarkable calculus weighing fifteen ounces, removed from a human subject by Mr. Harmer, a Norwich surgeon, in 1746. The patient was a man aged forty-eight, who ought to have been cut at eight years of age. He was robust, but intemperate. The Median operation, known as the Marion, was performed upon him on June 8th, Gooch being present. After entering through a common-sized wound, Mr. Harmer found it impossible either to remove or break the stone. He therefore desired Gooch to enlarge the wound, whilst he himself kept up a gentle extraction, and finally removed the stone. The patient recovered without a dangerous symptom, but the wound obstinately refusing to heal, the surgeon at last induced a favourite little dog to lick the parts. The application of the animal's moist tongue, and the subsequent use of soft dry linen was most soothing, and afforded the patient more relief than any surgical means. To the day of his death, in April, 1751, the man's canine friend acted as his surgeon. It would be interesting to know whether the dog long survived his patient, but the Museum records are too prosaic to concern themselves with that fact. However, here the stone is, a monstrous growth to have been where it actually was. Mr. Donne was the first surgeon appointed to the hospital, and there are some 170 of his cases in this collection. His reputation as a lithotomist was very high, and the rate of mortality amongst his cases was one in seven. One of his successful operations resulted in the removal of a curious mulberry-like stone, cut from a man named North, aged 36, in April, 1776. The cure was complete by August, and the stone weighs 2 oz. 8 scruples. Another celebrated cure, completed in three months after the operation, was that of Morse. He was also 36 years of age. The operation was performed in June, 1776, and the stone, which is wholly phosphatic, weighs 8 oz. Mr. Rigby, the father of Edward Rigby, the gynæcologist, was assistant surgeon for nineteen years, surgeon for four-and-twenty, and physician for seven years more—making a total of fifty years' connexion with the hospital—and operated during that time on 106 patients, with results which may be studied in the museum. Mr. Martineau was another successful stone-surgeon, operating, during the 50 years he was connected with the hospital, on 149 cases with a mortality of only one in eight. Mr. Dalrymple, the father of Mr. J. Dalrymple, the ophthalmologist, was surgeon for a quarter of a-century, and besides the records of his own work in the particular department of which we are now treating, this museum possesses another memorial of the family in a very fine collection of diseases of the eye, put up by his son, John, and presented by his brother, Donald Dalrymple. The most remarkable stone added to the collection during the time of Dalrymple is one weighing thirteen ounces, which was removed after the death of the patient, all attempts to extract it during life having failed. Among the most noteworthy of the long line of surgeons who have been connected with the hospital is Mr. J. G. Crosse, F.R.S., who was surgeon for twenty-seven years, and operated on some fifty cases. This number, comparatively small though it be, has yielded more scientific knowledge than a larger roll of cases would have returned in other hands, owing mainly to his habits of copious note-taking, and of critical commentary on all he saw and did. There is, in the museum, another miscellaneous collection, which contains among other things a section of Liston's vesical calculus, and a remarkable specimen of stone in the kidney—a uric acid deposit weighing no less than a pound and a-quarter. The traditions of Norwich Hospital are worthily sustained in the present day by Mr. Cadge, the senior surgeon—whose record of operations has now reached to more than three hundred, and whose recent lectures on "Stone in the Bladder," at the Royal College of Surgeons, are well remembered—and by Mr. T. W. Crosse.

This brief recapitulation of the men who have assisted in making Norwich famous in the world of surgical science is practically an account of the Hospital Museum. The deposits most frequently met with in the eastern counties are generally composed of pure uric acid, and are of intense hardness; and here, nine out of every ten of the stones present these characteristics. There are, however, many other items in the collection bearing upon the subject, amongst which the most noticeable is an old gorget which was used for lithotomy in a surgical era contemporaneous with that of Gooch's instruments before mentioned. There are also on the shelves, bottles containing the organs upon which the operations of lithotomy and lithotrity have been performed with ultimately fatal results, due in most cases to some disease of the kidneys One of the most frequent causes of death in the hospital many years ago was pyæmia, from which the institution suffered greatly, all attempts to abate it having failed. At length the whole system of nursing was changed, the entire charge was placed under the direction of a lady superintendent, and a revolution in management was established. Pyæmia disappeared as if by magic, and from that time—eleven or twelve years ago, in the days of the old hospital building—there have been no deaths from pyæmia, and the surgeons, in the words of Mr. Cadge, "have almost ceased to think of it." There are illustrations on the shelves of enlarged prostate glands, and a few splendid specimens of fungous and other tumours. Amongst some specimens of calculus of the kidneys is one stone 5½ by 6½ inches, composed of ammonio-phosphate of magnesia. The lay visitor, indeed, will marvel more and more at the long suffering of which diseased organs are capable, and of the extent to which a man may have a lithuric deposit, growing ever larger month by month in a most important organ, without for years being conscious of the fact.

Among other curiosities in the museum may be mentioned the skeleton of a dwarf named Pycroft, who was hanged for murder in 1820. The total height of the body is only 4 ft. 5 in., but the spine is quite an ordinary length, so that the tips of the hands only just come down to the hip joints. Lastly, there is the skull of Sir Thomas Browne, who, though he was born in London, was intimately associated with Norwich, where he died in 1682. The card which accompanies this exhibit contains, not a quotation from Browne's best-known work, *Religio Medici*, but a sentence chosen, not without a grim sense of humour, from his *Hydriotaphia*, "To be gnawed out of our graves, to have our skulls made drinking bowls, and our bones turned into pipes, to delight and sport our enemies, are tragical abominations escaped in burning burials."

